# *Acanthamoeba polyphaga mimivirus* Stability in Environmental and Clinical Substrates: Implications for Virus Detection and Isolation

**DOI:** 10.1371/journal.pone.0087811

**Published:** 2014-02-03

**Authors:** Fábio P. Dornas, Lorena C. F. Silva, Gabriel M. de Almeida, Rafael K. Campos, Paulo V. M. Boratto, Ana P. M. Franco-Luiz, Bernard La Scola, Paulo C. P. Ferreira, Erna G. Kroon, Jônatas S. Abrahão

**Affiliations:** 1 Universidade Federal de Minas Gerais, Instituto de Ciências Biológicas, Laboratório de Vírus, Belo Horizonte, Minas Gerais, Brazil; 2 URMITE CNRS UMR 6236– IRD 3R198, Aix Marseille Universite, Marseille, France; Nanyang Technical University, United States of America

## Abstract

Viruses are extremely diverse and abundant and are present in countless environments. Giant viruses of the *Megavirales* order have emerged as a fascinating research topic for virologists around the world. As evidence of their ubiquity and ecological impact, mimiviruses have been found in multiple environmental samples. However, isolation of these viruses from environmental samples is inefficient, mainly due to methodological limitations and lack of information regarding the interactions between viruses and substrates. In this work, we demonstrate the long-lasting stability of mimivirus in environmental (freshwater and saline water) and hospital (ventilator plastic device tube) substrates, showing the detection of infectious particles after more than 9 months. In addition, an enrichment protocol was implemented that remarkably increased mimivirus detection from all tested substrates, including field tests. Moreover, biological, morphological and genetic tests revealed that the enrichment protocol maintained mimivirus particle integrity. In conclusion, our work demonstrated the stability of APMV in samples of environmental and health interest and proposed a reliable and easy protocol to improve giant virus isolation. The data presented here can guide future giant virus detection and isolation studies.

## Introduction

Viruses are extremely diverse and abundant and are present in countless environments [1]. In some extreme ecosystems, viruses are the only known microbial predators, and they are powerful agents of gene transfer and microbial evolution [1]. Although viral genomes are ubiquitous in the biosphere, very little is known regarding the ecological roles of viruses in most ecosystems [2], [3]. In this context, large nucleocytoplasmic DNA viruses (NCLDVs) emerge as a fascinating research topic for virologists around the world. NCLDVs are frequently found in environmental samples, demonstrating their ubiquity and ecological impact [4]. There is still much to learn about NCLDV host-pathogen relationships and their impact on evolution, ecology and medicine [4].

*Acanthamoeba polyphaga mimivirus* (APMV), the prototype of the *Mimiviridae* family, was discovered in a hospital water cooling system in Bradford, England, during an outbreak of pneumonia [5]. APMV is an amoeba-associated virus with peculiar features, including a double-stranded DNA, ∼1.2 megabase (Mb) genome encoding proteins not previously observed in other viruses, such as aminoacyl-tRNA synthetases and DNA repair chaperones and enzymes, a >700 nm particle diameter and capsid-associated fibers [5], [6]. In 2008, a new giant virus named *A. castellanii mamavirus* (ACMV) was isolated from a cooling tower in Paris [7], [8]. Other known giant viruses include *Megavirus chiliensis*, isolated from Chilean ocean water [9]; *Lentille virus*, from the contact lens fluid of a patient with keratitis [10]; and *Moumouvirus*, isolated from cooling tower water [11].

*Acanthamoeba* is believed to be the natural host of *Mimiviridae* [5], though there is evidence of mimivirus replication in vertebrate phagocytes. These amoebae are ubiquitous and have been isolated from aquatic environments, soil, air, hospitals and contact lens fluid. Amoebas are part of vertebrates’ normal microbiota, and they are extremely resistant to pH variations, high temperatures and disinfectants [12], [13]. Considering the ubiquity of *Acanthamoeba*, giant viruses could hypothetically be found everywhere. Metagenomic analysis demonstrated the presence of mimivirus-like sequences in many aquatic environments [12], [14]. There are few data on APMV in animal tissues or its hypothetical role as pneumonia agent, although a recent metagenomic study found mimivirus DNA in bovine serum [15]. Free-living amoebae may potentially propagate pathogens in hospital environments, and hospitalized patients would represent a group of risk for amoeba-associated pneumonia agents, including APMV [5], [16]–[18].

The ubiquity of APMV DNA in the environment but the lack of information about the ecological – and medical – impact of these viruses warrants their isolation and characterization. Research groups trying to “prospect” giant viruses in the laboratory [9], [11], [19] have difficulty recovering these viruses from environmental samples; there is also no standard protocol for the optimization of isolation techniques [19], [20]. Most giant virus prospecting studies rely on direct co-culture of samples with amoebas to propagate viruses. However, the pre-enrichment of environmental samples can be useful for viral isolation, as demonstrated by the discovery of megavirus [9]. In this study, we verified the stability of APMV in hospital and environmental substrates and validated an enrichment protocol for APMV isolation. Our results suggest that the enrichment protocol improves APMV detection from different substrates but does not modify some viral genetic and biological features. Our study may be useful in future giant virus prospecting studies.

## Materials and Methods

### APMV Preparation

APMV particles were isolated and purified from infected amoebae as previously described [16]. Briefly, *Acanthamoeba castellanii* (ATCC 30234) were grown in 75 cm^2^ cell culture flasks (Nunc, USA) in PYG (peptone-yeast extract-glucose) medium supplemented with 7% fetal calf serum (FCS, Cultilab, Brazil), 25 mg/ml Fungizone (amphotericin B, Cristalia, São Paulo, Brazil), 500 U/ml penicillin and 50 mg/ml gentamicin (Schering-Plough, Brazil). After reaching confluence, the amoebas were infected with APMV and incubated at 37°C until the appearance of cytopathic effects. APMV-rich supernatants from the infected amoeba were collected and filtered through a 0.8-micron (Millipore, USA) filter to remove amoeba debris. The viruses were then purified using a Gastrografin gradient (45–36–28%) [5], suspended in PBS and stored at −80°C.

### Virus Titration

Samples were serially diluted (1/10) in PYG medium, and 100 µl was inoculated onto 10^5^ amoeba seeded in a 96-well Costar® microplate (Corning, NY) on the previous day (8 wells per dilution, 200 µl final volume). Plates were incubated for 2–4 days at 32°C to determine the highest dilution that led to amoebal lysis (TCID_50_/ml) [26].

### Virus Recovery from Substrates, Enrichment and Stability Tests

**Figure 1:**
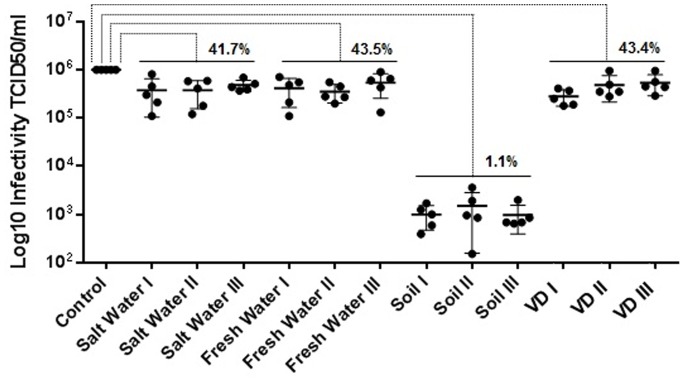
APMV recovery from samples after 1 hour. 10^6^ TCID_50_ of purified APMV were added to salt water (10 ml), fresh water (10 ml), topsoil (1 g) and three different brands of VD substrates, and after one hour, virus recovery was evaluated by titration in *A*. *castellanii*. The fresh water and soil samples were collected from three different Brazilian biomes: Amazon and Mata Atlântica, two highly biodiverse rainforests, and Cerrado, a savanna-like biome. The salt water samples were collected from 3 points on the coast of South and Southeast Brazil, for a total of five samples per substrate per biome. The results are the means+SD of an experiment performed in quintuplicate.

To test the APMV recovery from the assayed substrates, a total of 10^6^ TCID_50_ of purified APMV was re-suspended in phosphate buffered saline (PBS) and added to autoclaved salt water (10 ml), fresh water (10 ml) and topsoil (1 g). The fresh water and soil samples were collected from three different Brazilian biomes: Amazon and Mata Atlântica, two very biodiverse rainforests, and Cerrado, a savanna-like biome. The salt water samples were collected from 3 points on the coast of South and Southeast Brazil, for a total of five samples per substrate per biome (Figure 1). The water and land collections were performed with permission of Instituto Chico Mendes (ICM) – protocol numbers: 34293-1 and 33326-2. The field studies did not involve endangered or protected species. Considering the hypothetical role of APMV as pneumonia agent associated with prolonged mechanical ventilation the viral stability in ventilator devices was evaluated. Three different brands of sterile ventilator device (tube) (VD) were used to test APMV recovery (five quadrants of 2×2 cm per brand) [21]. In this case, 10 µl of viral suspension was added to VD quadrants with or without BAV, which were maintained in sterile Petri dishes. After one hour, all the samples were titrated in *A*. *castellanii* by the TCID_50_ method as described [26]. All the environmental and VD samples were previously tested for APMV DNA and/or infectious particles [5].

APMV stability was analyzed for 12 months in the substrates described above. Samples were maintained in 15 ml tubes at room temperature (Figure S1). Every month, 500 µl aliquots from each substrate were collected, serial diluted in PBS and titrated in amoebae. The viral titer was adjusted to the total assayed volume. The sample enrichment protocol was adapted from Arslan et al. (2011). Briefly, 500 µl of samples were added to 450 ml water-rice medium (40 grains of rice per liter of water) and kept in the dark at room temperature for 5 days. Following this incubation, 5000 pathogen-free amoebas were added to the samples, and 5000 more amoebas were added after twenty days. The samples were titrated in amoebae after thirty days and then every subsequent month for one year. All samples were used in real-time PCRs targeting the conserved hel gene (primers: 5′ACCTGATCCACATCCCATAACTAAA3′ and 5′GGCCTCATCAACAAATGGTTTCT3′). Samples DNA were extracted by Phenol-ChloroformThe real-time PCR was performed with a commercial mix Power SYBr Green (Applied Biosystems, USA), primers (4 mM each) and 1 µl sample in reaction of 10 µl final volume. All reactions were performed in a StepOne thermocycler: 95°C-10 min, 40 cycles- 95°C-15 s/60°-15 s, followed by a dissociation step (specific Tm = 73°C).

### Transmission Electron Microscopy

*A. castellanii* were infected at an MOI of 10. Uninfected amoebae were used as controls. At 7 h post-infection, amoebae were washed twice with PBS and fixed with 2.5% glutaraldehyde type 1 (Merck, Germany) for one hour at room temperature. The amoebae culture was dislodged with a cell scraper and centrifuged at 900×g for 5 min. Ultrathin sections were prepared [5] by the Centro de Microscopia, UFMG, Brazil.

### One-step Growth Curves

To compare the replication of APMV before and after enrichment, one-step growth curve assays were performed. *A. castellanii* were infected at an MOI of 10. The infectivity was measured by TCID50 after 25 hours (0 to 25 hours) by observing the CPE in amoeba.

### Sequencing Analysis

The hel and GlcT genes were amplified by PCR from purified APMV and from samples after enrichment. The hel and GlcT genes were chosen since they have been used as markers in phylogenetic or in evolutionary studies [5], [9], [11]. The primers were designed based on APMV sequences available in GenBank (Accession NC 014649). The amplicons were directly sequenced in both orientations and in triplicate (Mega-BACE sequencer, GE Healthcare, Buckinghamshire, UK). The sequences were aligned with Genbank references with ClustalW and were manually aligned using MEGA software version 4.1 (Arizona State University, Phoenix, AZ, USA).

## Results

### APMV is Stable for Long Periods in Different Substrates

To evaluate APMV stability in environmental and hospital substrates, an initial experiment analyzed the recovery of APMV from each substrate. A total of 10^6^ TCID_50_ of purified APMV was re-suspended in phosphate buffered saline (PBS) and added to previously autoclaved salt water (10 ml), fresh water (10 ml) and topsoil (1 g). The fresh water and soil samples were collected from three different Brazilian biomes (Amazon, Cerrado and Mata Atlântica), and the salt water samples were collected from 3 points on the coast of South and Southeast Brazil, for a total of five samples per substrate per biome (Figure 1). Three different brands of sterile ventilator device (tube) (VD) were used to test APMV recovery (five quadrants of 2×2 cm per brand) [21]. After one hour, all the samples were titrated in *A*. *castellanii*. The average values from quintuple replicates showed >40% virus recovery from salt water, fresh water and VD samples, regardless of the biome or brand. In contrast, viral recovery from soil was approximately 1% in all samples (Figure 1).

**Figure 2:**
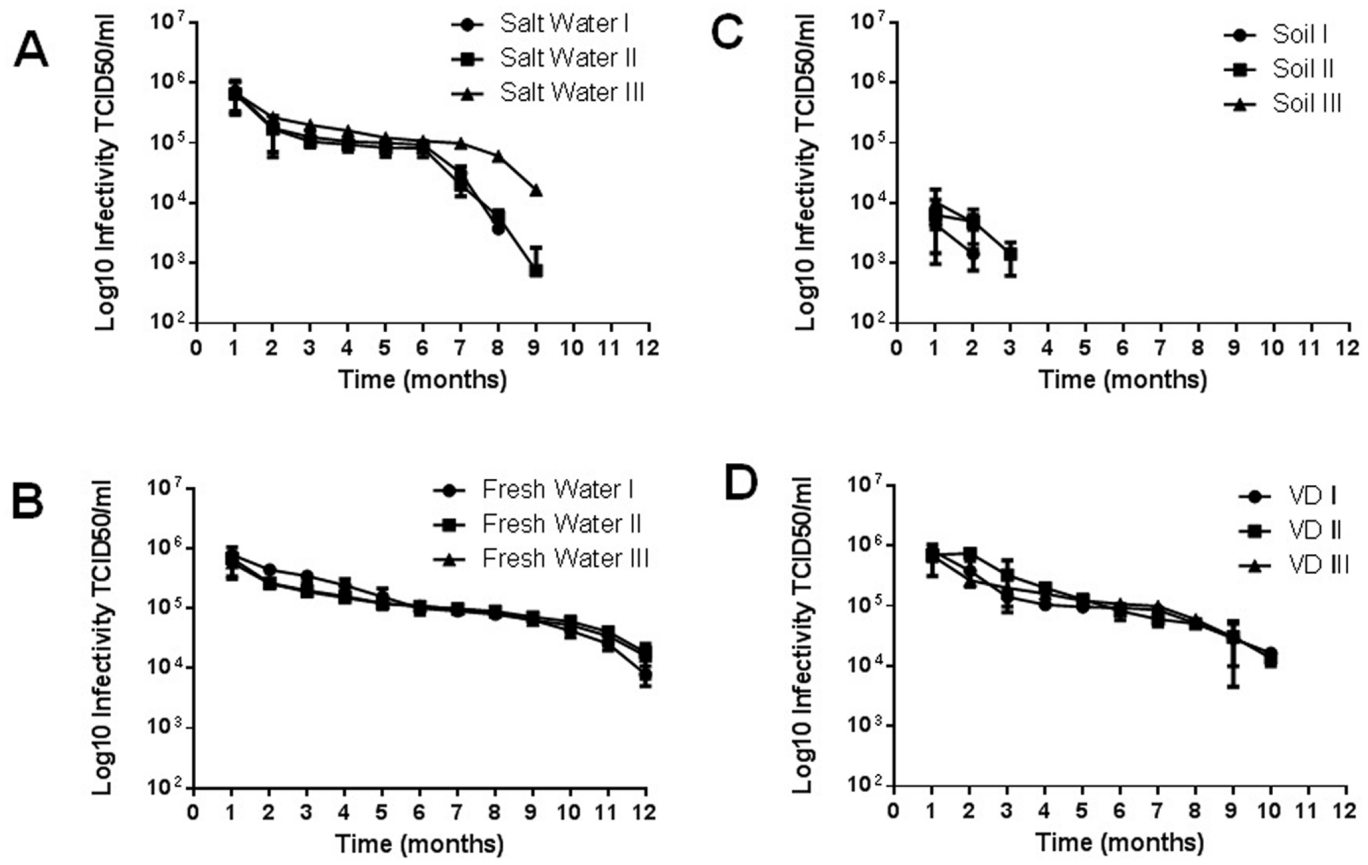
APMV stability in different substrates. To evaluate the long-term stability of APMV in different substrates for one year, 10^6^ TCID_50_ of purified APMV were added to salt water, soil, fresh water and VD substrates, which were maintained in 15 ml tubes at room temperature. At monthly intervals, the samples were titrated in amoebae. (A) Salt water; (B) Fresh water; (C) Soil; (D) VD. The results are the means+SD of an experiment performed in quintuplicate. I, II and III represent independent experiments performed with samples collected from distinct locations.

The stability of APMV was analyzed for 12 months in each substrate. Samples were prepared and maintained in 15 ml tubes at room temperature. Relative humidity and temperature were monitored through the experiment (relative humidity average max-72%; min 56%/temperature average max-25°C; min-19°C) (Figure S1). At monthly intervals, aliquots from each substrate were collected, serially diluted in PBS and titrated in amoebae. Titration assays revealed the long-term stability of APMV in salt and fresh water and VD, as infectious particles were detected in each substrate after 9, 12 and 10 months, respectively (Figure 2A, 2B, 2D). In general, the viral titers decreased less than 1 log until the 6^th^ month, after which time virus titers decreased eventually to undetectable levels. APMV could be isolated from soil samples until the third month, but the viral titer was decreased by >2 log after the first month (Figure 2C).

### Enrichment of APMV from Different Substrates

**Figure 3:**
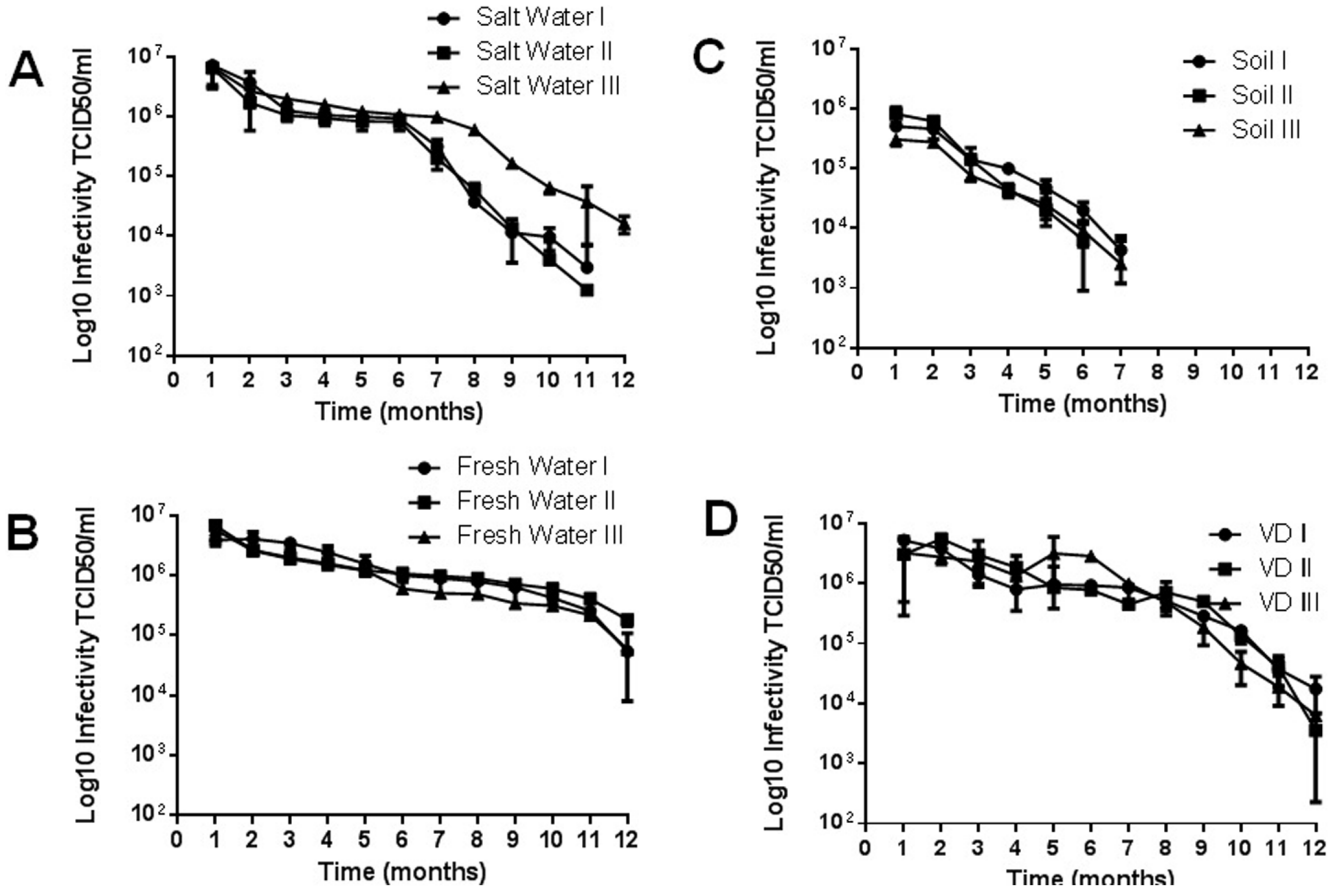
APMV isolation in different substrates after enrichment. As above, substrates were inoculated with APMV (10^6^ viral particles) and then enriched. At one-month intervals, the samples were titrated in amoebae. (A) Salt water; (B) Fresh water; (C) Soil; (D) VD. The results are the means+SD of an experiment performed in quintuplicate. I, II and III represent independent experiments performed with samples collected from distinct locations.

While recovery of APMV depended on the substrate in which the virus was immersed, recovery was never complete, and the viral titer decreased over time in all substrates. To verify that an enrichment protocol following sample collection could improve viral recovery, we prepared substrates as described above containing 10^6^ TCID_50_ APMV and then enriched the viruses in these samples [9]. Briefly, 500 µL each sample was added to 450 ml water-rice medium (40 grains of rice per liter of water) and stored in the dark at room temperature for 5 days. Following this incubation, 5000 amoebas were added to the samples, and 5000 more amoebas were added after twenty days. After thirty days, the samples were titrated in amoebae; samples were then titrated again monthly for one year. The enrichment process improved APMV recovery from all the analyzed substrates. APMV was detected after one year in salt and fresh water (Figure 3A and 3B), seven months in soil (Figure 3C) and one year in VD samples (Figure 3D). When compared with the results shown in Figure 2, the overall viral titer and the viral recovery time were increased after the enrichment. In enriched soil samples, virus could be detected up to seven months; this duration was much improved from the unenriched maximum detection time of three months.

### APMV Stability in VD Containing BAL Samples

**Figure 4:**
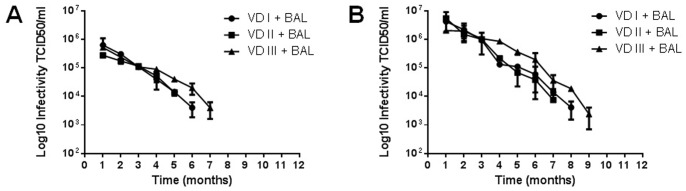
APMV stability in VD enriched after BAL addition. Purified APMV (10^6^ viral particles) were added to VD samples, and subsequently, BAL samples were added. The BAL-VD samples were or were not enriched and maintained at room temperature. The samples were titrated in amoebae monthly. (A) Samples without enrichment; (B) Enriched samples. The results are the means+SD of an experiment performed in quintuplicate.

Detection of APMV in VD is clinically relevant and could establish this virus as a human pathogen [16]. One factor that might impact viral detection in VD is the presence of bronchoalveolar lavage (BAL). To verify if BAL impairs APMV recovery from VD samples, 10^6^ TCID_50_ of APMV were added to VD containing BAL, which were then enriched. While BAL generally reduced APMV recovery (Figure 4A), the enrichment protocol increased the initial viral titers and allowed the isolation of APMV up to the 9^th^ month of the experiment (Figure 4B).

### Real-time PCR of APMV in Environmental and Hospital Substrates

As shown above, the optimal recovery APMV results from pre-enrichment of substrates. Viral isolation from environmental samples is vital for the characterization of new giant viruses, but it is not absolutely required for some ecological, evolutionary or epidemiological studies. Molecular viral detection, e.g., PCR, is faster, cheaper and less laborious than viral isolation. To verify that enrichment improves APMV detection by PCR, the samples described in the previous experiments were used as templates for real-time PCRs designed to detect APMV helicase (hel). APMV DNA was detected in all water and VD samples, with or without enrichment. APMV DNA was detected up to the eleventh month in non-enriched salt water samples and up to the twelfth month in enriched samples. In soil samples, APMV DNA was detected up to five months and nine months in unenriched and enriched samples, respectively. Enrichment also improved APMV detection in VD containing BAL samples, with a detection time shift from the eighth to the eleventh month (Table 1).

**Table 1:** APMV detection by real-time PCR in samples with or without enrichment.

	APMV detection by PCRa,b	
Sample	With no Enrichment (months)	Post – Enrichment (months)
Salt Water	1^st^ to 11^th^	1^st^ to 12^th^
Fresh Water	1^st^ to 12^th^	1^st^ to 12^th^
Soil	1^st^ to 5^th^	1^st^ to 9^th^
VD	1^st^ to 12^th^	1^st^ to 12^th^
VD+BAL	1^st^ to 8^th^	1^st^ to 11^th^

a Real-time PCR – Helicase gene;

b Results presented considering all replicates.

### APMV Biology, Morphology and DNA Sequences are not Changed by Enrichment

**Figure 5:**
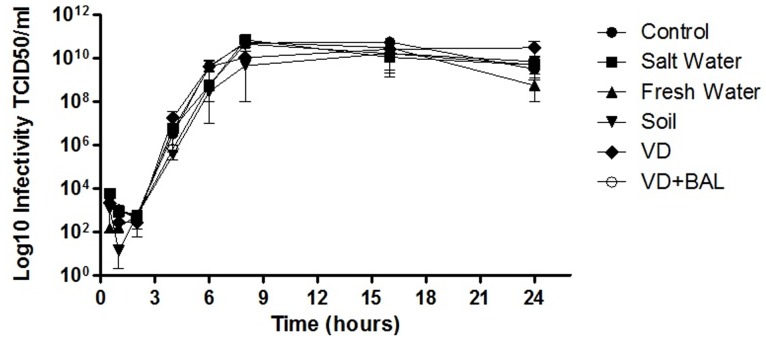
APMV one-step growth curves with or without enrichment. Purified APMV (10^6^ viral particles) was added to salt water, soil, fresh water and VD substrates, which were then enriched. After viral isolation from each substrate, *A. castellanii* were infected at an MOI of 10. The infectivity was measured by TCID_50_ after 25 hours (0 to 25 hours) by observing CPE in amoeba. The results are the means of experiments performed in duplicate.

Virus isolation in laboratory conditions could promote genotype and/or phenotype changes by artificial selection. Boyer et al. (2011) showed dramatic changes in APMV genomes, replication and morphology after sub-culturing APMV in a germ-free amoeba host. To evaluate possible changes in APMV caused by the enrichment process, morphological, virological and genetic assays were performed. No differences were observed in one-step growth curves of virus recovered from all assayed samples, suggesting that no biological modifications occurred during enrichment (Figure 5). These samples were also observed by electron microscopy, and no morphological changes were detected when compared to APMV images previously published [5], [7]. (Figure S2). In addition, the sequences of the APMV hel and GlcT genes from enriched samples were 100% identical to the APMV reference sequences in Genbank (Figures S3 and S4).

### Enrichment Applicability Tests

To test the applicability of the enrichment protocol, water samples were collected from 2 urban lakes and from a river in Southeast and North Brazil. A total of 475 samples of 5 ml each were collected from the water surface. Aliquots of the samples were enriched or directly inoculated onto *A. castellanii* monolayers for virus isolation. In parallel, real-time PCRs to detect APMV were performed.

From the 475 samples, six giant viruses were isolated with the enrichment protocol (Table 2). These viruses induced cytopathic effects in amoebas, including cell rounding and lysis, and were positive in PCR assays. In contrast, only one of the six virus isolates was propagated by direct inoculation of the water samples in amoebas. Sequencing of hel gene confirmed that all virus isolates belonged to the *Megavirales* order (data not shown).

**Table 2:** Enrichment applicability tests: detection of giant viruses with no and post-enrichment by viral isolation and real-time PCR.

		Positive samples [total of positive (%)]^a^		
Sample	Total of samples (n)		With no Enrichment	Post – Enrichment
Lake 1	325		1 (0.3%)	2 (0.6%)
Lake 2	88		0 (0%)	2 (2.27%)
River	35		0 (0%)	2 (5.71%)
**Total**	**475**		**1 (0.21%)**	**6 (1.2%)**

a Isolation in *A. castellani* monolayer+positivity in hel gene real-Time PCR.

## Discussion

Virus isolation has technical limitations, and classical protocols based on filtration have delayed the detection of giant viruses [22]. Another problem for giant virus isolation is their limited known host range, which restricts the cellular systems that can be used for *in vitro* culture [22]. Thus, most information on giant viruses comes from environmental metagenomic studies and not from virus isolation [22]. This approach must be made with extreme caution, as comparing fragmented environmental sequences with those found in databases can result in false assumptions about giant virus complexity and HGT events [14]. Isolation of these viruses is imperative for understanding their roles in the environment and as potential vertebrate pathogens.

The ubiquity of amoebae implies the probable ubiquity of *Mimiviridae*, which has been confirmed by indirect viral detection methods. Despite their remarkable abundance, giant viruses are not easily isolated. Many of these viruses are unknown, and their stability in environmental samples is not determined. Furthermore, there are few described approaches for viral isolation or protocols that favor viral isolation from complex substrates. Our investigation estimates APMV stability in several relevant substrates and shows that enrichment prior to isolation optimizes viral stability and recovery from any substrate.

In our first analysis, APMV recovery was reduced at one hour after its addition to different substrates. Although viral recovery from fresh water, salt water or VD was approximately 40% of the initial viral load, viral recovery from soil samples was much lower, representing less than 2% of the input viruses (Figure 1). The reasons for these recovery ranges include microbial influence and physical or chemical characteristics of the substrate. These factors interfere with survival through viral particle aggregation and virucidal activity. Particularly for APMV, viral aggregation is important since through their capsid surrounded by fibrils can occurs easily aggregation and influence in the filtration process required for viral isolation [12], [23], [24]. Following these results, we measured the stability of APMV in the substrates mentioned above for twelve months. Virus was recovered from fresh water at all time points tested, and virus was recovered from salt water, VD and soil until the ninth, tenth and third months of the experiment, respectively (Figure 2). Viral titers dropped over time in all substrates due to the reasons stated above.

In theory, an enrichment protocol could increase the number of giant viruses in a sample (viral replication) and, thus, their likelihood of recovery. This protocol favors the proliferation of heterotrophic organisms that are consumed by amoebas, and these amoebas are used for giant virus replication. After establishing the stability of APMV in different substrates, we added an enrichment protocol prior to viral isolation. The enrichment improved the method sensitivity, as viruses were isolated at later time points compared with samples without enrichment. Furthermore, enrichment increased virus yields (Figure 3).

Among the analyzed substrates, VD is particularly interesting for the hypothetical pathogenic aspect of *Mimiviridae*, as APMV has been detected in pneumonia patients. Devices such as VD are sources of nosocomial infections and could be sources for giant virus infections as well. We showed that APMV had long-term stability in VD and that the pre-enrichment improved viral detection. In a hospital setting, VDs would most likely be filled with clinical specimens that could interfere with APMV stability and recovery. To test this possibility, we added BAL to VDs before adding APMV and then determined viral recovery and stability with or without enrichment. BAL affected APMV stability; APMV was recovered from BAL-containing VDs only up to seven months without enrichment. In this case, enrichment allowed APMV recovery at nine months (Figure 4). BAL decreased APMV stability but did not completely neutralize the virus, so these samples were still potentially infectious. As shown previously, enrichment improved APMV recovery from BAL-containing VDs and was thus useful for isolating the giant virus from these samples.

APMV in the enriched or unenriched samples was also quantified by real-time PCR, which is more sensitive than cell culture isolation and is also improved by enrichment (Table 1). While isolation needs viable viral particles in the sample, PCR detection needs viral DNA from any viral particles, whether viable, inactivated or defective. Thus, genomic studies can also benefit from the enrichment protocol described in this paper.

In allopatric conditions, successive passages of APMV in amoebae drastically reduce the viral genome, resulting in morphological and genetic changes [25]. To verify that enrichment protocol did not modify the original virus, we compared one-step growth curves from viruses obtained after enrichment to the prototype virus. The curves were similar, suggesting that the enrichment did not cause any biological alterations (Figure 5). We also verified the absence of morphological and genetic changes in APMV by electron microscopy and sequencing. All viruses were similar in size and had the characteristic APMV fibers and internal membranes (Figure S2). Analysis of hel and GlcT revealed that enrichment did not alter these genes, as they were identical to reference sequences (Figure S3).

In conclusion, our work determines the stability of APMV in samples of environmental and clinical interest and proposes a reliable and easy protocol to improve giant virus isolation. This protocol can be used for giant virus prospecting studies.

## Supporting Information

Figure S1 **Temperature and relative humidity averages during the 12 experimental months.**
(TIF)

Figure S2 **APMV do not exhibit changes in viral morphology after enrichment.** Purified APMV (10^6^ viral particles) were added to salt water, soil, fresh water and VD substrates, which were then enriched. After viral isolation from each substrate, *A. castellanii* were infected at an MOI of 10 and analyzed by EM at 8 hours post-infection. A, B, C and D: APMV recovery from enriched salt water, fresh water, soil and VD, respectively.
(TIF)

Figure S3 **APMV do not show changes in the GlcT gene after enrichment.** PCR for the GlcT gene was performed using enriched APMV samples as templates, and the resulting amplicon was sequenced. The DNA sequences were aligned with APMV reference sequences from Genbank using the ClustalW method and were manually aligned using MEGA software version 4.1 (Arizona State University, Phoenix, AZ, USA).
(TIF)

Figure S4 **APMV do not show changes in the hel gene after enrichment.** PCR for the hel gene was performed using enriched APMV samples as templates, and the resulting amplicon was sequenced. The DNA sequences were aligned with APMV reference sequences from Genbank using the ClustalW method and were manually aligned using MEGA software version 4.1 (Arizona State University, Phoenix, AZ, USA).
(TIF)
