# Supplementary figures and images for: Acanthamoeba polyphaga mimivirus Stability in Environmental and Clinical Substrates: Implications for Virus Detection and Isolation
Source: PLoS One. 2014 Feb 3;9(2):e87811. doi: 10.1371/journal.pone.0087811 (PMC3912154; doi:10.1371/journal.pone.0087811)

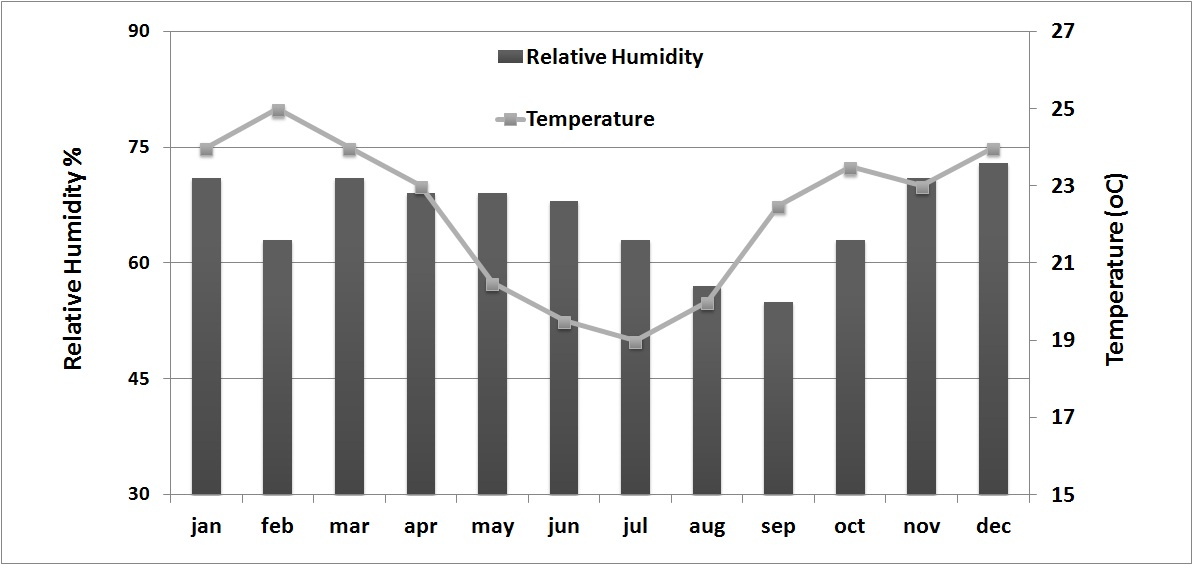

Supplement: Figure S1 — Temperature and relative humidity averages during the 12 experimental months. (TIF) [file pone.0087811.s001.tif]

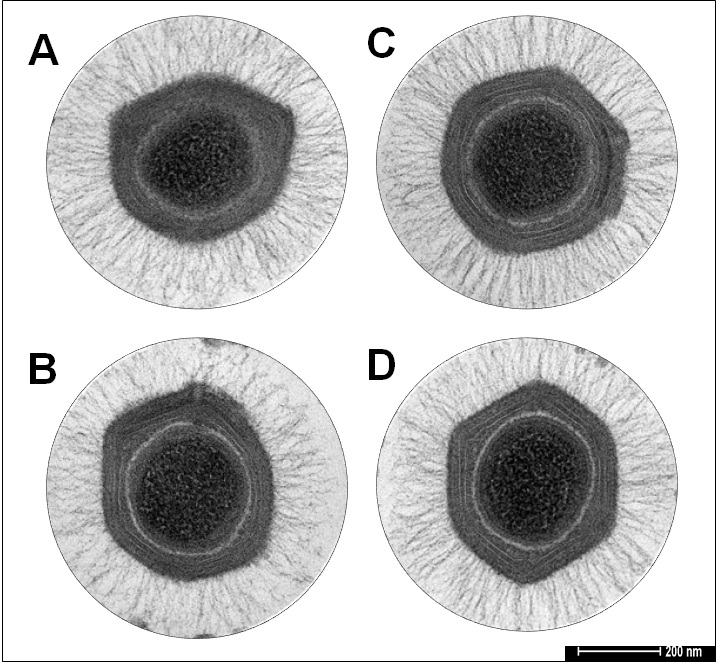

Supplement: Figure S2 — APMV do not exhibit changes in viral morphology after enrichment. Purified APMV (106 viral particles) were added to salt water, soil, fresh water and VD substrates, which were then enriched. After viral isolation from each substrate, A. castellanii were infected at an MOI of 10 and analyzed by EM at 8 hours post-infection. A, B, C and D: APMV recovery from enriched salt water, fresh water, soil and VD, respectively. (TIF) [file pone.0087811.s002.tif]

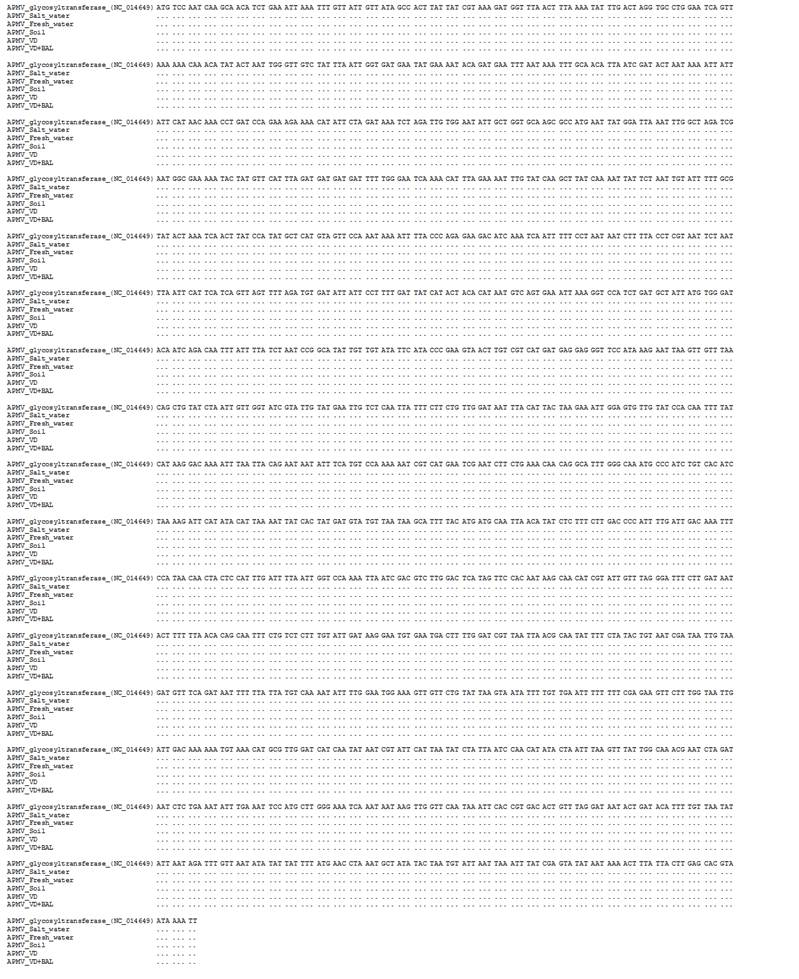

Supplement: Figure S3 — APMV do not show changes in the GlcT gene after enrichment. PCR for the GlcT gene was performed using enriched APMV samples as templates, and the resulting amplicon was sequenced. The DNA sequences were aligned with APMV reference sequences from Genbank using the ClustalW method and were manually aligned using MEGA software version 4.1 (Arizona State University, Phoenix, AZ, USA). (TIF) [file pone.0087811.s003.tif]

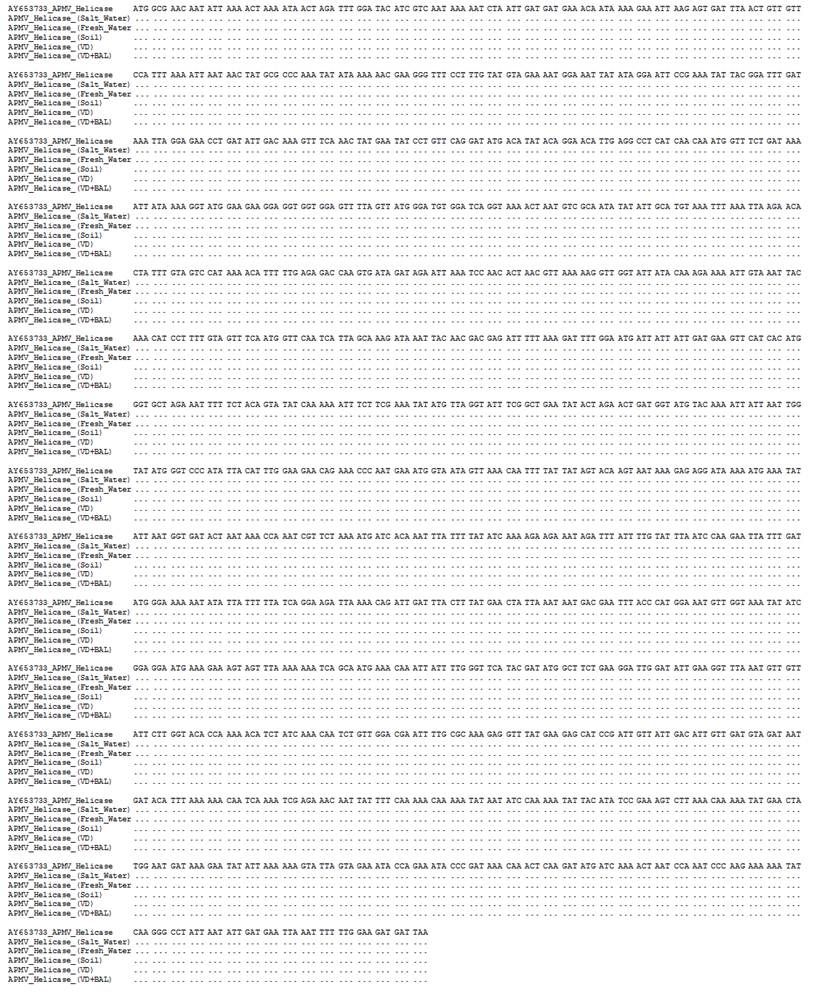

Supplement: Figure S4 — APMV do not show changes in the hel gene after enrichment. PCR for the hel gene was performed using enriched APMV samples as templates, and the resulting amplicon was sequenced. The DNA sequences were aligned with APMV reference sequences from Genbank using the ClustalW method and were manually aligned using MEGA software version 4.1 (Arizona State University, Phoenix, AZ, USA). (TIF) [file pone.0087811.s004.tif]
